# Interaction between the ADAMTS-12 metalloprotease and fibulin-2 induces tumor-suppressive effects in breast cancer cells

**DOI:** 10.18632/oncotarget.1690

**Published:** 2014-01-12

**Authors:** Tania Fontanil, Susana Rúa, María Llamazares, Angela Moncada-Pazos, Pedro M. Quirós, Olivia García-Suórez, Jose A. Vega, Takako Sasaki, Yamina Mohamedi, Manuel M. Esteban, Alvaro J. Obaya, Santiago Cal

**Affiliations:** ^1^ Departamento de Bioquímica y Biología Molecular, Facultad de Medicina, Universidad de Oviedo, Asturias, Spain; ^2^ IUOPA, Instituto Universitario de Oncología del Principado de Asturias, Spain; ^3^ German Cancer Research Center (DKFZ) Division Signal Transduction and Growth, Heidelberg, Germany; ^4^ William Dunn School of Pathology, University of Oxford, Oxford, United Kingdom; ^5^ Morfología y Biología Celular (grupo SINPOS), Facultad de Medicina, Universidad de Oviedo, Asturias, Spain; ^6^ Department of Biochemistry, Faculty of Medicine, Oita University, Japan; ^7^ Biología Funcional, Facultad de Medicina, Universidad de Oviedo, Asturias, Spain

**Keywords:** extracellular matrix, metalloprotease, ADAMTS, fibulin, breast cancer

## Abstract

Balance between pro-tumor and anti-tumor effects may be affected by molecular interactions within tumor microenvironment. On this basis we searched for molecular partners of ADAMTS-12, a secreted metalloprotease that shows both oncogenic and tumor-suppressive effects. Using its spacer region as a bait in a yeast two-hybrid screen, we identified fibulin-2 as a potential ADAMTS-12-interacting protein. Fibulins are components of basement membranes and elastic matrix fibers in connective tissue. Besides this structural function, fibulins also play crucial roles in different biological events, including tumorigenesis. To examine the functional consequences of the ADAMTS-12/fibulin-2 interaction, we performed different *in vitro* assays using two breast cancer cell lines: the poorly invasive MCF-7 and the highly invasive MDA-MB-231. Overall our data indicate that this interaction promotes anti-tumor effects in breast cancer cells. To assess the in vivo relevance of this interaction, we induced tumors in nude mice using MCF-7 cells expressing both ADAMTS-12 and fibulin-2 that showed a remarkable growth deficiency. Additionally, we also found that ADAMTS-12 may elicit pro-tumor effects in the absence of fibulin-2. Immunohistochemical staining of breast cancer samples allowed the detection of both ADAMTS-12 and fibulin-2 in the connective tissue surrounding tumor area in less aggressive carcinomas. However, both proteins are hardly detected in more aggressive tumors. These data and survival analysis plots of breast cancer patients suggest that concomitant detection of ADAMTS-12 and fibulin-2 could be a good prognosis marker in breast cancer diagnosis.

## INTRODUCTION

Extracellular matrix (ECM) components and their interactions are essential for homeostasis of tissues and provide structural support to animal cells [1-2]. These components also play crucial roles on cell dynamic behavior and act as modulators of intercellular communication [3-4]. Among them, fibulins family consists of seven secreted glycoproteins that share a globular domain at the carboxy-terminus, known as fibulin module, which is preceded by tandem calcium-binding Epithelial Growth Factor (cbEGF) domains [5-7]. Amino-terminal region displays the highest structural variability. Functionally fibulins serve as a scaffold for different ECM components including proteoglycans [8], fibronectin [9], collagen [10], fibrillin [11] or laminin [12]. In addition to this structural role, fibulins can also participate in complex biological processes including cell migration, adhesion and proliferation [13], and mediate cell signaling events [14-15].

Fibulin-2 illustrates an example for the contribution of fibulins to those processes. In fact, fibulin-2 is recognized as a multifunctional binding protein due to its ability to bridge diverse ECM components [8, 16]. For instance, fibulin-2 participates in the assembling of the laminin network in the basement membrane through the binding of laminin-1 and laminin-5 to other matrix proteins [17]. Also, binding of nidogen to fibulin-2 contributes to the formation of ternary complexes with type IV collagen, perlecan and fibulin-1 [18]. Moreover, fibulin-2 promotes cell-ECM contacts by binding to β3 integrins [19]. These and other examples [16, 20] highlight the relevance of fibulin-2 in the maintenance of extracellular structures such as basement membranes, as well as contacts between cells and ECM. Complexity of functions of fibulin-2 is also evident in pathological conditions such as cancer [13, 21]. In this regard, pro-tumor effects have been associated to this fibulin in pancreatic cancer cells as consequence of its interaction with the transmembrane type I glycoprotein MUC4 [22]. However, a growing number of studies point out the anti-tumor properties of fibulin-2. For instance, this fibulin acts as an anti-angiogenic factor in nasopharyngeal carcinoma [23]; *FBLN2*, the human gene coding for fibulin-2, is found epigenetically silenced in B cell acute lymphoblastic leukemia [24]; and loss of fibulin-2 expression facilitates tumor progression in breast cancer cells [25]. These findings reveal the importance of fibulin-2 in tumor development. Nonetheless, the molecular mechanism by which this fibulin promotes or inhibits tumorigenesis is unclear at present.

ADAMTS-12 is a secreted metalloprotease [26] and different studies have suggested a role for this metalloprotease in tissue remodeling and cell migration or adhesion [27-28]. Moreover, ADAMTS-12 acts as a tumor-suppressor enzyme. Thus, this enzyme hinders anti-tumor effects in Madin-Darby canine kidney (MDCK) cells treated with hepatocyte growth factor [29]. Furthermore, *ADAMTS12* gene promoter is hypermethylated in colorectal carcinomas [30]. Phenotypic analysis of the *Adamts12*-deficient mouse has confirmed the tumor-protective role of this enzyme [31]. By contrast, ADAMTS-12 has been also associated to trophoblastic cell invasion by modulating the expression and function of α_v_β_3_ integrin [32]. Exploring the mechanisms that link ADAMTS-12 to tumorigenesis would help to minimize pro-tumor properties and thereby enhance anti-tumor effects of this metalloprotease.

Here we show that ADAMTS-12 and fibulin-2 are two interacting proteins. Functional consequences of this interaction were evaluated using different cell-based assays. Two breast cancer cell lines, MCF-7 and MDA-MB-231, were employed attending to the anti-tumor role of fibulin-2 [25] and expression profile of *ADAMTS12* [33] in this type of tumor. Immunohistochemical detection of fibulin-2 and ADAMTS-12 was also performed using a human breast cancer tissue array. Main conclusion is that ADAMTS-12/fibulin-2 interaction potentiates anti-tumor effects in breast cancer cells.

## RESULTS

### ADAMTS-12 and fibulin-2 are two interacting proteins

**Figure 1:**
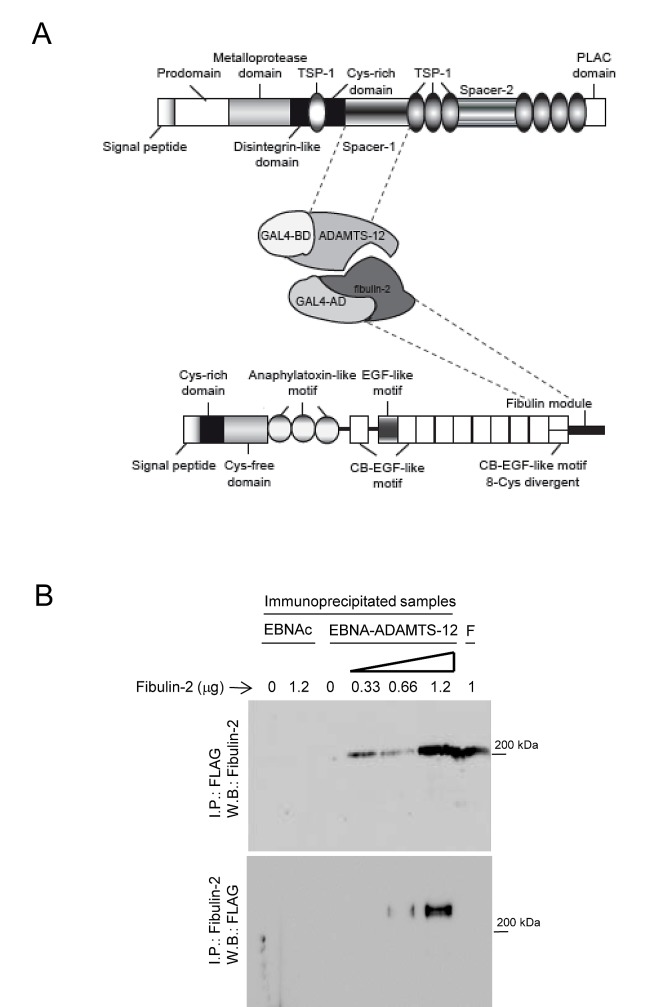
ADAMTS-12 and fibulin-2 are two interacting proteins (A) Schematic illustration of the interacting regions between ADAMTS-12 and fibulin-2 determined by yeast two-hybrid screen. TSP-1, thrombospondin-1-like domain; Cys-rich domain, cysteine-rich domain; PLAC, protease-lacunin; RGD, Arginine-Glycine-Aspartic Acid; EGF, ephitelial growth factor; CB-EGF, Calcium-binding EGF; BD, binding domain; AD, activating domain. (B) Western blot analysis of immunoprecipitated samples. Top, 293-EBNA control cells extracts (EBNAc) or 293-EBNA cell extracts producing ADAMTS-12-FLAG (EBNA-ADAMTS-12) were incubated with the indicated amounts of fibulin-2, immunoprecipitated with using anti-FLAG M2 affinity gel and further detection was performed with an anti-fibulin-2 antibody. Bottom, cell extracts were immunoprecipitated with an anti-fibulin-2 antibody and detection was performed with an anti-ADAMTS-12 antibody. F indicates that 1 μg of fibulin-2 was loaded in these lanes. Molecular weight marker is indicated on the right.

Different fragments corresponding to the ADAMTS-12 exosites, including thrombospondins and spacer regions, were employed as baits to screen for molecular partners of the metalloprotease using yeast two-hybrid assays. Among them, a fragment corresponding to spacer-1 allowed to select different clones containing inserts that according to BLAST analysis at NCBI (www.ncbi.nlm.nih.gov) matched with extracellular matrix proteins such as NELL2, connective tissue growth factor or agrin. Interestingly, one of these clones, with a 630-bp insert, corresponded to nucleotides 3360 to 3990 of human fibulin-2 cDNA (GenBank™ accession number X82494.1). Conceptual translation of this region showed that the sequence matched with the carboxyl-end region of fibulin-2, including the final 88 amino acids of the fibulin module (Fig.1A). Taking in account that an interaction between fibulin-1 and ADAMTS-1 had been previously described [34], we decided to explore the functional consequences of a new interaction between an ADAMTS metalloprotease and a fibulin. As a first approach to validate this interaction we examined a potential co-immunoprecipitation of both proteins. To this end, we employed 293-EBNA cell extracts containing ADAMTS-12 tagged with a FLAG epitope. Different amounts of fibulin-2 were added to these extracts and immunoprecipitation was performed using an anti-FLAG M2 affinity gel. Immunoprecipitates were visualized by Western blot using an anti-fibulin-2 antibody (Fig. 1B). An immunoreactive band corresponding to fibulin-2 was detected in those cell extracts containing ADAMTS-12 incubated with the fibulin. However this band was not detected without incubation with fibulin-2 or when the same immunoprecipitation procedure was applied to extracts of EBNA control cells (EBNAc) (Fig. 1B). Conversely, cell extracts were also immunoprecipitated with an anti-fibulin-2 antibody and immunoblotted with an anti-FLAG antibody. In this case, an immunoreactive band corresponding to ADAMTS-12 was detected in two samples containing fibulin-2, but not in control samples (Fig. 1B). These results strongly suggest that fibulin-2 physically binds to ADAMTS-12. Considering this, we decided to examine whether fibulin-2 could act as a cofactor for the metalloprotease, similarly to what occurs with fibulin-1 and ADAMTS-1 [34]. However, presence of fibulin-2 did not increase the low aggrecanase activity of ADAMTS-12 using a peptide corresponding to the aggrecan IGD domain as substrate (Supplementary Fig. S1).

### Interaction between fibulin-2 and ADAMTS-12 reduces breast cancer cell invasion and migration

**Figure 2:**
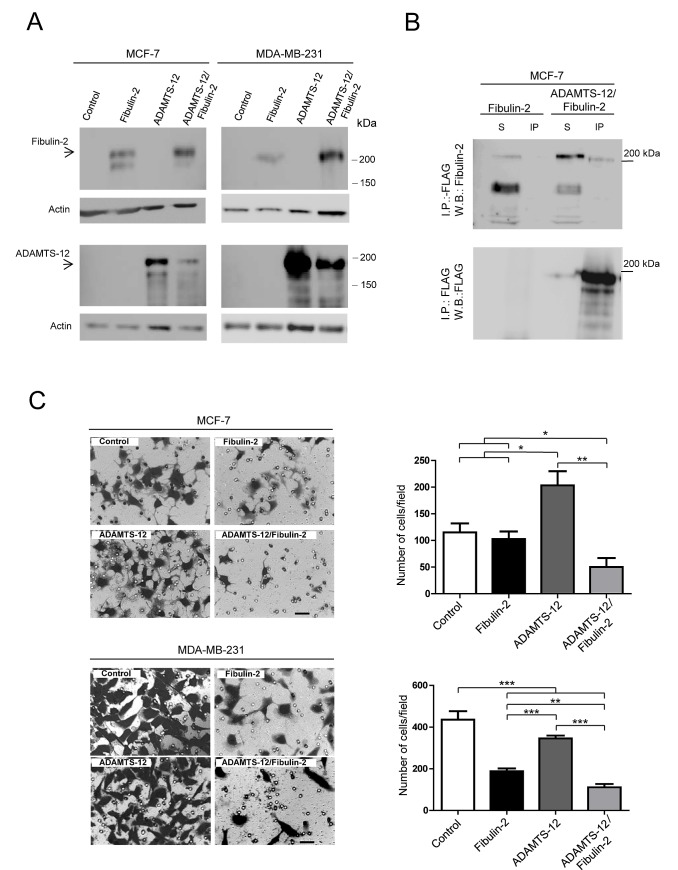
Selection of MCF-7 and MDA-MB-231 stable transfectants and invasion assay (A) Western blot analysis of MCF-7 and MDA-MB-231 producing exogenous fibulin-2, ADAMTS-12 or both proteins simultaneously (ADAMTS-12/fibulin-2). These cells are referred as (fb), (ts) and (fb/ts) respectively in the body text. Control, cells transfected with an empty vector. Top, detection with an anti-fibulin-2 antibody. Bottom, detection with an anti-FLAG (ADAMTS-12) antibody. Samples were run in separated gels due to the similar molecular weights of fibulin-2 and ADAMTS-12. Actin was used as a loading control. Molecular weight markers are indicated on the right. (B) Immunoprecipitation of MCF-7 cell extracts producing fibulin-2 or both fibulin-2 and ADAMTS-12 using anti-FLAG M2 affinity gel. S, cell extracts. IP, immunoprepitated. Top, detection with an anti-fibulin-2 antibody. Bottom, detection with an anti-FLAG (ADAMTS-12) antibody. (C) Cell invasion assay using Matrigel-coated invasion chambers. Representative microscopic pictures of MCF-7 (top) and MDA-MB-231 (bottom) cells producing exogenous fibulin-2, ADAMTS-12 or both proteins simultaneously. Cells transfected with an empty vector were used as control. Cells that reached the lower surface were counted and graphically represented. Scale bar: 50 μm.

Next, we wanted to assess the functional consequences of the interaction between ADAMTS-12 and fibulin-2. To this end, we transfected MCF-7 and MDA-MB-231 cells with the full-length cDNAs for *ADAMTS12* and *FBLN2*. Production of recombinant fibulin-2 (fb), ADAMTS-12 (ts) or both proteins simultaneously (fb/ts) was analyzed by Western blot (Fig. 2A and supplementary Fig. S2). Prior to functional analysis, we proved that presence of these extracellular proteins did not affect cell proliferation (Supplementary Fig. S3), and that the interaction between fibulin-2 and ADAMTS-12 also took place in MCF-7-(fb/ts) cells (Fig. 2B). Then, to analyze the role of this interaction in cell invasion, cells were allowed to invade using Matrigel-coated invasion chambers (Fig. 2C). This assay showed that both MCF-7-(fb/ts) and MDA-MB-231-(fb/ts) cells exhibited a very low invasive potential in comparison with control cells (63 %, and 76 % reduction respectively), or with cells producing only fibulin-2 (58 % and 47 % reduction). Similarly to what has been previously reported [25], the highly invasive MDA-MB-231 cell line producing fibulin-2, MDA-MB-231-(fb), remarkably reduced its invasive potential comparing to control cells (55 % reduction, Fig. 2C). However, in the case of the poorly invasive MCF-7 no differences were observed between control and MCF-7-(fb) cells. By contrast, both MDA-MB-231-(ts) and MCF-7-(ts) showed a higher invasive ability than MDA-MB-231-(fb) and MCF-7-(fb) respectively (Fig. 2C).

**Figures 3:**
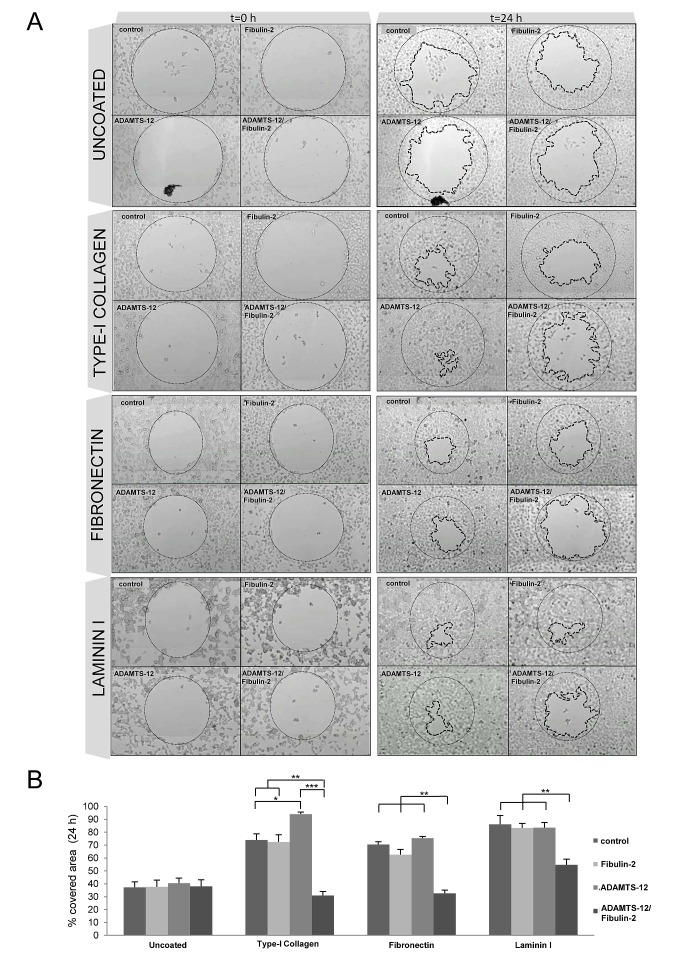
Interaction between fibulin-2 and ADAMTS-12 hinders migration of MCF-7 cells on ECM components (A) MCF-7-(fb) (fibulin-2), MCF-7-(ts) (ADAMTS-12), and MCF-7-(fb/ts) (ADAMTS-12/fibulin-2) were allowed to migrate in uncoated wells or wells coated with type-I collagen, fibronectin or laminin I. MCF-7 cells transfected with an empty vector were used as control. Pictures of starting (t=0 h) and final (t=24 h) times are included. Starting point is indicated with a thin dotted line and final point with a thick dotted line. (B) Graphical representation of covered area after 24 h from three independent experiments.

Interaction between fibulin-2 and ADAMTS-12 also affected cell migration. As can be seen in Fig. 3A, MCF-7-(fb/ts) cells did not show differences (37 % covered area) respecting MCF-7-(ts) (39 %), MCF-7-(fb) (37 %) or control cells (37 %) when migration was evaluated after 24 h using uncoated wells (supplementary videos 1 to 4). However, a significant reduction in migratory capacity of MCF-7-(fb/ts) cells was observed when these cells were grown in wells coated with fibronectin, laminin I, or type-I collagen (supplementary videos 5 to 8). Thus, 35 % and 37 % of area was covered by MCF-7-(fb/ts) cells in wells coated with type-I collagen and fibronectin, respectively. Nonetheless, MCF-7-(ts), MCF-7-(fb) and control cells covered 91 %, 70 % and 71 % on type-I collagen, and 75 %, 62 % and 70 % on fibronectin respectively. In the case of laminin I, MCF-7-(fb/ts) cells covered 54 % whereas MCF-7-(ts), MCF-7-(fb) and control cells covered around 85 % (Fig. 3B). Similar experiments performed with MDA-MB-231 cells revealed that fibulin-2/ADAMTS-12 interaction also hindered the ability of these cells to migrate, but differences were not so pronounced between MDA-MB-231-(fb) and MDA-MB-231-(fb/ts) as in MCF-7 cells (Supplementary Fig. S4). Altogether, these data support that ADAMTS-12 fibulin-2/ interaction hampers both invasion and migration of breast cancer cells *in vitro*, but also that ADAMTS-12 could exert pro-tumor effects in the absence of fibulin-2.

### Combined expression of FBLN2 and ADAMTS12 reduces mammosphere formation

**Figure 4:**
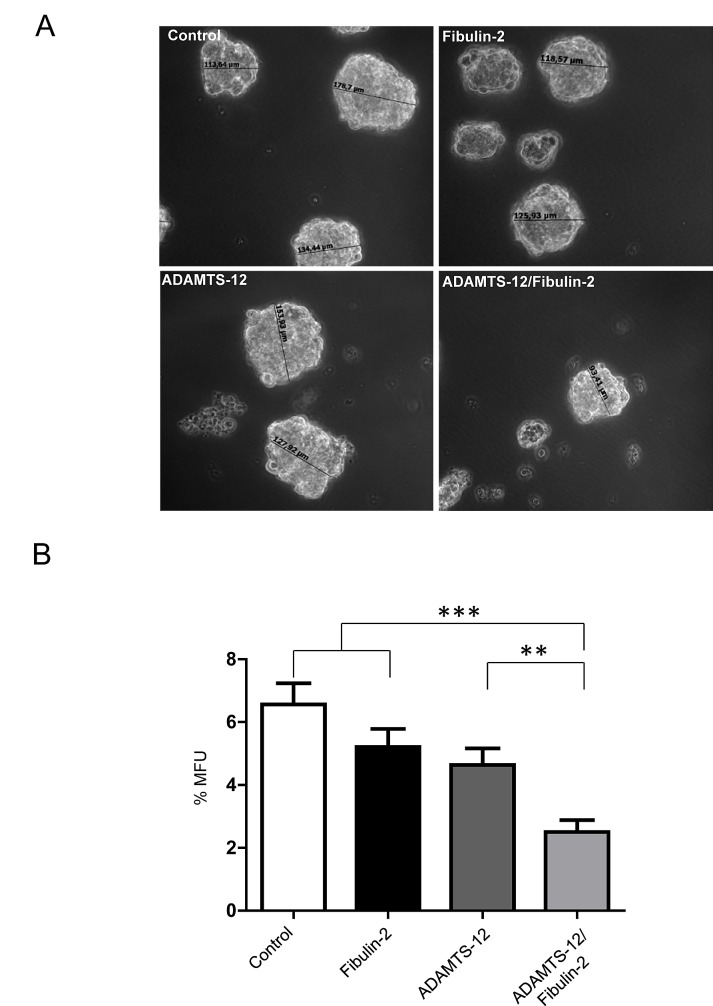
Simultaneous production of fibulin-2 and ADAMTS-12 reduces self-renewal of mammosphere-forming units in MCF-7 cells (A) Representative images of mammospheres derived from MCF-7-(fb) (fibulin-2), MCF-7-(ts) (ADAMTS-12), and MCF-7-(fb/ts) (ADAMTS-12/fibulin-2). Control, MCF-7 cells transfected with an empty vector. Sizes of some mammospheres are indicated. (B) Mammospheres were dissociated and passaged at a density of 20 cell/well in 96-well plates, and MFUs were counted and calculated as a percentage of mammospheres formed from the number of cell seeded.

3D culture system mimics the natural tissue environment of the cell. In the case of breast cancer cells, this culture system allows mammosphere formation and reflects the ability of these cells for *in vitro* self-renewal. Therefore we examined whether ADAMTS-12 and fibulin-2 could also alter the mammosphere-forming ability of breast cancer cells. To this end, 4 x 10^4^ MCF-7 cells were plated in 6-well plates and mammospheres were visualized after 7 days. MCF-7-(fb/ts) showed a smaller number and mammospheres size than MCF-7-(fb), MCF-7-(ts) or MCF-7 control cells (Fig. 4A and 4B). To quantify the mammosphere forming units (MFUs), cells were dissociated using trypsin and passaged at a density of 20 cells/well through a 96-well plate. 7 days later, MFUs were calculated and MCF-7-(fb/ts) showed 3.2-fold decrease of MFUs compared to MCF-7 control cells, and 2-fold decrease compared to MCF-7-(fb) and MCF-7-(ts) cells (Fig. 4B). In the case of MDA-MB-231 cells, exogenous expression of ADAMTS-12 and fibulin-2 also caused a decrease in MFUs compared to MDA-MB-231-(ts) cells (2-fold) and to MDA-MB-231 control cells (2.5-fold). However, difference between MDA-MB-231-(fb/ts) and MDA-MB-231-(fb) was slight (Supplementary Fig. S5A and S5B). Overall, these results indicate that fibulin-2 and ADAMTS-12 affect ability of breast cancer cells to form mammospheres.

### Interaction between fibulin-2 and ADAMTS-12 inhibits tumor growth in vivo

**Figure 5:**
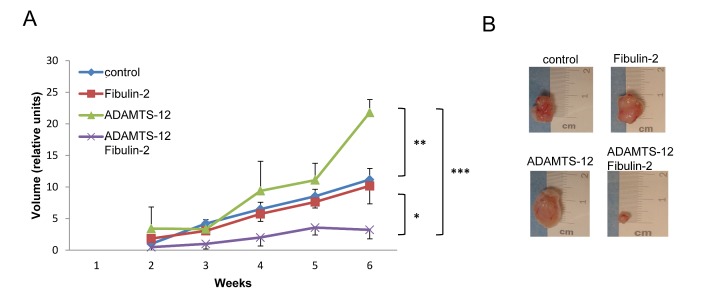
Presence of fibulin-2 and ADAMTS-12 inhibits growth of subcutaneous tumors (A) Nude mice were injected with MCF-7-(fb) (fibulin-2), MCF-7-(ts) (ADAMTS-12), MCF-7-(fb/ts) (ADAMTS-12/fibulin-2) or control MCF-7 cells, and tumors growth were weekly measured. (B) Representative subcutaneous tumors at the time of sacrifice (6 weeks).

To evaluate the effect of *ADAMTS12* and *FBLN2* expression *in vivo*, MCF-7-(fb), MCF-7-(ts) or MCF-7-(fb/ts) cells were subcutaneously injected in the flank of nude mice. Control MCF-7 cells were injected in the opposite flank and the evolution of tumor growth was followed by 6 weeks. MCF-7-(fb/ts) cells displayed a significantly reduced tumor growth rate when comparing to MCF-7-(fb), MCF-7-(ts) or MCF-7 control cells (Fig. 5). Thus, at the time of sacrifice, tumor volume derived from MCF-7-(fb/ts) cells was, on average, 25 % smaller than those derived from MCF-7-(fb) and MCF-7 control cells, and 90 % smaller than those derived from MCF-7-(ts) cells. These results indicate that the interaction between fibulin-2 and ADAMTS-12 may also promote anti-tumor effects *in vivo.*

### Inverse correlation of ADAMTS12 and FBLN2 expression with breast cancer stage

**Figure 6:**
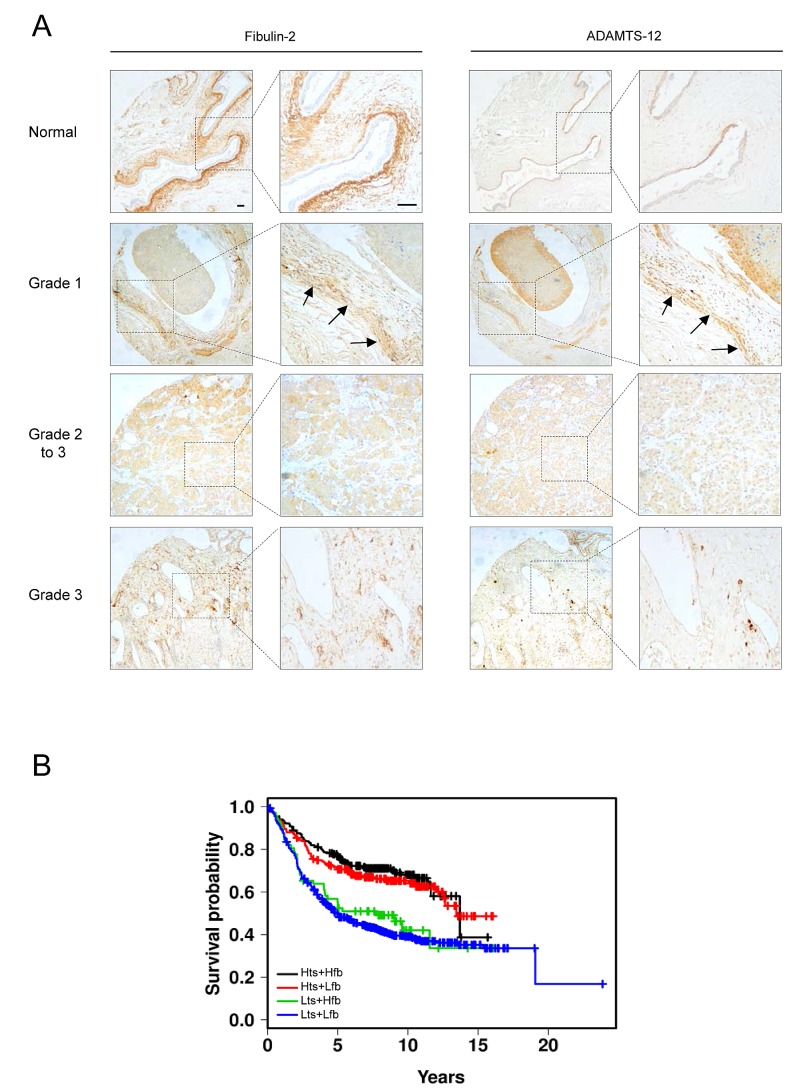
FBLN2 and ADAMTS12 expression analysis in breast cancer samples (A) representative tissue images for detection of ADAMTS-12 and fibulin-2 in healthy breast tissue and breast carcinoma samples. Arrows indicate detection of ADAMTS-12 and fibulin-2 in grade 1 breast carcinoma. Scale bar, 200 μm. (B) Kaplan-Meier survival plots showing a better outcome of breast patients with high expression level of both *ADAMTS12* and *FBLN2* (Hts+Hfb). Plots for high *ADAMTS12* and low *FBLN2* (Hts+Lfb); low *ADAMTS12* and high *FBLN2* (Lts+Hfb); and low *ADAMTS12* and low *FBLN2* (Lts+Lfb) are also included.

To determine clinical relevance of ADAMTS-12 and fibulin-2 in breast cancer, we performed an immunohistochemical analysis of a tissue array containing 16 samples of normal and breast cancer tissues. A strong staining for fibulin-2 was mainly detected in periductal connective tissue in normal breast (Fig. 6A). ADAMTS-12 was also detected in epithelia duct cells but showing a weaker staining. A moderate to strong expression of both fibulin-2 and ADAMTS-12 was detected in connective tissue surrounding tumor in grade 1 or 1 to 2 breast carcinoma (n=5). However, weak or absent staining was observed in 10 out 11 (91 %) samples of grade 2 and 3 breast carcinoma for both fibulin-2 and ADAMTS-12. These data point to an inverse correlation between *FBLN2* and *ADAMTS12* co-expression and histophatological tumor grade.

We next assessed the association between *ADAMTS12* and *FBLN2* expression with clinical outcome using data available at www.kmplot.com. Kaplan-Meier curves revealed that a combined high expression of both *ADAMTS12* and *FBLN2* correlated with the best prognosis in breast cancer patients (Hts+Hfb, Fig. 6B) when compared to patients expressing low levels of both genes (Lts+Lfb) (*p* < 0.001). This analysis also showed that simultaneous high *ADAMTS12* and low *FBLN2* expressions (Hts+Lfb) associated to better outcome. However, high expression levels of *FBLN2* combined with a low expression of *ADAMTS12* (Lts+Hfb) showed a slight but not significant increase of survival rate (Fig. 6B). These data suggests that factors other than combined expression of these two genes could modulate the pro or anti-tumor activities of both fibulin-2 and ADAMTS-12.

## DISCUSSION

ECM regulates almost all cellular behavior and multiple regulatory mechanisms to ensure that production and remodeling of ECM components are normal during organ development [1, 35]. Altered ECM can trigger cell signals or molecular interactions that promote cell survival or proliferation thereby contributing to cancer development [36]. Hence a major challenge in ECM biology is to understand the role of its components in normal and pathological processes. In this regard, a growing number of studies emphasize the multiple roles of fibulins in development and homeostasis of tissues [16]. Beyond this structural role, both tumor-suppressive functions and oncogenic activities have also been related to these glycoproteins [21]. Diverse mechanisms may underlie these apparently contradictory roles in cancer, including their interactions with other extracellular matrix components [13].

In the present work, using a yeast two-hybrid screening, we have found that fibulin-2 is a new partner of the ADAMTS-12 metalloprotease. Interaction between these two secreted proteins was confirmed by co-immunoprecipitation. Functional consequences of this interaction were evaluated by different cell-based assays using two human breast cancer cell lines, the poorly invasive MCF-7 and the highly invasive MDA-MB-231. None of them express fibulin-2 [25] or ADAMTS-12 endogenously [30-31], hence we have selected clones producing ADAMTS-12, fibulin-2 or both proteins simultaneously. Exogenous expression of these proteins did not alter cell growth rates. However, results obtained from other cell-based assays point to two important findings. First, anti-tumor properties associated to fibulin-2 in breast cancer [25] are enhanced by its interaction with ADAMTS-12, especially in MCF-7 cells. Thus, production of both proteins remarkably reduced MCF-7 cells ability to invade in Matrigel-coated invasion chambers. This interaction also decreased the percentage of mammosphere-forming units, and strongly inhibited cell migration on fibronectin or laminin, two extracellular matrix components that associate to fibulin-2 [17]. Additionally, MCF-7-(fb/ts) cells displayed a reduced tumor growth when were subcutaneosuly injected in the flank of nude mice. Data obtained using MDA-MB-231 cells indicated that differences were less marked between MDA-MB-231-(fb/ts) and MDA-MB-231-(fb). In this regard, it has been previously found that pro-tumor properties of MDA-MB-231 cells are especially hampered by fibulin-2. In fact, exogenous expression of fibulin-2 in this cell line reduced speed of wound-healing closure and decreased cell invasion *in vitro* [25]. Moreover, fibronectin-induced migration of MDA-MB-231 cells is markedly inhibited by fibulin-1 [37], the closest molecular partner of fibulin-2. Consequently, both fibulin-1 and -2 may inhibit oncogenic properties of breast cancer cells. Mechanisms underlying these effects are not yet known, but association with other ECM components could also influence pro-tumor or anti-tumor activities of fibulins.

The second relevant finding is that ADAMTS-12 could promote pro-tumor properties when is produced in breast cancer cells in the absence of fibulin-2. Exogenous expression of the metalloprotease not only increased invasion and migration capacities of MCF-7 cells *in vitro* but also produced significantly higher tumor volumes in nude mice. Contrary to this, we had previously found that exogenous expression of *ADAMTS12* in A549 cells inhibited tumor growth *in vivo* [29]. An explanation for these apparent contradictory results is that A549 cells express fibulin-2 [14]. Thus, it can be speculated that exogenous ADAMTS-12 may interact with fibulin-2 produced by A549 cells and thereby inducing the anti-tumor effect observed. A previous report also supported the pro-tumor activity of this metalloprotease since its exogenous expression potentiated invasive capacity of the human placental choriocarcinoma cell line JEG-3 [32]. Moreover, exogenous ADAMTS-12 reduced the ability of JEG-3 cells to bind to collagen-1 and our data indicate that presence of ADAMTS-12 increases migration on collagen-1 of MCF-7 cells. Interestingly, an enhanced stromal collagen-1 deposition correlates with breast cancer metastasis [38]. In this scenario and in the absence of fibulin-2, ADAMTS-12 could contribute to tumor progression by increasing the migratory capacity of breast tumor cells on collagen-1. By contrast, several studies have highlighted the anti-tumor role of this metalloprotease, including the phenotypic characterization of *Adamts12*-deficient mouse [31]. Different mechanisms may cause the anti-tumor effects of ADAMTS-12. For instance, thrombospondin type-1 motifs at carboxy-terminal region of the protease influence its antimetastatic potential. However this enzyme undergoes proteolytic processing events that could modify this effect [29]. Some of these properties resemble those previously described for ADAMTS-1. Thus, this metalloprotease was initially catalogued as a tumor-suppressor factor due to the angio-inhibitory properties related to the thrombospondin type-1 domains located at the carboxy-terminal region [39]. Furthermore, a decreased expression of ADAMTS-1 in breast cancer favors breast cancer migration and invasion [40]. Nonetheless, ADAMTS-1 can also generate anti-angiogenic peptides through the digestion of thrombospondin-1[41] or endorse the recruitment of fibroblasts involved in tumor growth, an effect also associated to an increased deposition of collagen-1 [42]. Moreover, ADAMTS-1 interacts with other member of the fibulin family, fibulin-1 [34], which in this case acts as a cofactor of the metalloprotease. Hence, fibulin-1 could potentiate degradation of ECM components by ADAMTS-1 and thereby facilitating tumor progression. We explored the possibility that fibulin-2 could be a regulatory factor of the metalloprotease activity. Given that ADAMTS-12 displays low aggrecanase activity *in vitro* [29], we evaluated the digestion of a peptide corresponding to the human *aggrecan* interglobular domain. However, our results indicated that the presence of fibulin-2 did not potentiate the cleavage of this peptide by ADAMTS-12. Despite of this, it cannot be ruled out that this fibulin could act as an enhancer or inhibitor of ADAMTS-12 enzymatic activity in other circumstances considering the limited number of known *in vivo* substrates for this enzyme.

Immunohistochemical staining revealed that ADAMTS-12 was mainly detected in the stroma of grade 1 breast carcinomas, similar to what had been previously reported in colorectal cancer [30]. Staining pattern of fibulin-2 showed an equivalent situation. However, both proteins are hardly detected in grade 2 and 3 breast carcinomas. Thus, it can be speculated that a potential interaction between these two proteins may happen in less aggressive breast cancers and that this interaction could form part of a stromal response aimed to block cancer progression. Kaplan-Meier survival plots also suggested a potential tumor-suppressive role of the concomitant high expression of both *ADAMTS12* and *FBLN2*. However, some results of this work also suggest a pro-tumor activity of ADAMTS-12 in the absence of fibulin-2. In consequence, pro or anti-tumor activities of ADAMTS-12 could also be influenced by factors other than interaction with fibulin-2.

Interaction between ADAMTSs and fibulins raises new questions about the role of these proteins in tumor progression. For instance, these interactions may underlie the molecular mechanisms by which some tumor-protective metalloproteases exert their anti-tumor activities. In this work we show that ADAMTS-12/fibulin-2 interaction induces anti-tumor effects in breast cancer cells and our data also suggest that combined detection of both proteins could be a good prognosis marker in breast cancer patients. Further exhaustive functional and mechanistic studies will help finding out the precise role of ADAMTS-12 and fibulin-2 in relation to tumorigenesis, including the importance of the ADAMTS-12/fibulin-2 molar ratio as an indicator of potential invasiveness of breast cancer cells. Additionally, tumor susceptibility assays using mice lacking both *Adamts12* and *Fbln2* genes would be necessary to understand in depth the significance of this interaction not only in breast cancer but also in tumors from different origins.

## MATERIALS AND METHODS

### Yeast Two-hybrid Screening

The Matchmaker Two-hybrid System and a human fetal kidney library (prey vectors) containing a sequence enconding amino acids 768-881 of the GAL4 protein (AD) were purchased from Clontech. Screening process was performed following manufacturer´s recommendations. Briefly, a cDNA fragment encoding amino acids 702-813 of human ADAMTS-12 (GenBank™ accession number AAI31734) was PCR amplified using the primers 5´-AAACATATGCAGACTGTGAGAAAGAT GTT-3´ and 5´-TATGTCGACTGGATTGTGTACTCATA CTT-3´and inserted between the NdeI and SalI sites of the pGBKT7 vector to make a bait vector encoding a GAL4-BD-ADAMTS-12^702-813^ chimeric protein. Both bait and prey vectors were introduced into *Saccharomyces cerevisiae* AH109 cells and selection of double transformants was performed on synthetic minimal medium lacking leucine, tryptophan and histidine. Colonies were restreaked, and prey plasmids rescued, transfected into *Escherichia coli* DH5α and selected on LB medium containing 100 μg/mL ampicillin. Identification of the prey inserts was performed by direct sequencing.

### Human cell lines, plasmids and transfection

MCF-7 and MDA-MB-231 breast cancer cells, obtained from ATCC, and 293-EBNA cells producing exogenous ADAMTS-12 containing a FLAG epitope [29] were routinely maintained in DMEM medium containing 10 % heat-inactivated fetal bovine serum and 100 U/mL penicillin and 50 μg/mL streptomycin. pCEP-TS12 vector [29] and the full-length cDNA clon HK9 [8] were employed to overproduce ADAMTS-12 and human fibulin-2 respectively. These vectors were transfected into MCF-7 and MDA-MB-231 cells using Lipofectamine reagent (Life Technologies) as recommended by the manufacturer. Recombinant ADAMTS-12 and fibulin-2 were obtained as previously described [8, 29]. Cells stably expressing *ADAMTS12* (ts), *FBLN2* (fb) or both simultaneously (fb/ts), or transfected with an empty vector were selected in the presence of 1.5 μg/mL puromycin (Sigma-Aldrich).

### Western blotting and immunoprecipitation

For western blot analysis, proteins were resolved by 8 or 12 % polyacrylamide gel electrophoresis, transferred to a nitrocellulose membrane and subsequently probed with the indicated antibodies. Primary antibodies used for detection of ADAMTS-12 were H-142 (Santa Cruz Biotech) or anti-FLAG-M2 (Sigma-Aldrich), and for detection of fibulin-2 was H-250 (Santa Cruz Biotech). Anti-actin antibody was from Sigma-Aldrich. Immunoreactive proteins were visualized using HRP-peroxidase labeled anti-rabbit or anti-mouse secondary antibody and the ECL detection system (Pierce). Relative expression levels were quantified and normalized to actin levels within the same blot using the Image J software. For immunoprecipitation, MCF-7-(fb/ts) were resuspended in lysis buffer (20 mM Tris-HCl pH 8.0, 150 mM NaCl, 5 mM iodoacetamide, 0.5 mM CaCl_2_, 0.5 mM MgCl_2_, 0.02 % NaN3, 1mM AEBSF and 1 % Triton X-100), containing one complete protease inhibitor cocktail tablet (Roche Molecular Biochemicals) per 50 mL buffer. Protein concentration was quantified using the BCA Protein Assay Kit from Pierce Technology. To immunoprecipite ADAMTS-12, cell extracts were incubated with anti-FLAG M2 affinity gel (Sigma-Aldrich) following manufacturer´s instructions. To immunoprecipitate fibulin-2, we used H-250 antibody and the Protein G Immunoprecipitation kit (Sigma-Aldrich), following manufacturer´s instructions. MCF-7 cells extract producing fibulin-2 was used as a negative control. In the case of EBNA-293 cells producing ADAMTS-12, cell extracts were incubated with the indicated amounts of fibulin-2 prior to the immunoprecipitation step. EBNA cells transfected with an empty vector (EBNAc) were used as negative control.

### Enzyme activity

ADAMTS-12 enzymatic activity was evaluated using a 10xHis tagged peptide corresponding to the interglobular (IGD) domain of aggrecan as substrate at 0.016 μg/μl (rhAggrecan G1-IGD-G2, R&D Systems). As indicated, different amounts of fibulin-2 were added to the reaction. Reactions were performed in 20 μL for 4 hours in the buffer previously described [29]. Degradation by ADAMTS-4 (R&D Systems) was employed as positive control. Peptide degradation was examined by western blot using an anti-His antibody (Quiagen).

### Cell proliferation

Cell proliferation was measured using the CellTiter 96 Non-radiactive Cell Proliferation Assay kit purchased from Promega. 10^3^ cells were seeded in 96-well plates and six replicates per condition and time point were assessed. Cell proliferation rates were determined on three (MCF-7) or four (MDA-MB-231) consecutive days using an automated microtiter plate reader Power Wave WS (BioTek).

### Invasion assays

*In vitro* invasion potential was evaluated using 24-well Matrigel-coated invasion chambers with a 8 μm pore size (BD Biosciences). For MCF-7 cells, 2.5 x 10^4^ cells were allowed to migrate for 96 h using 10 % foetal bovine serum as a chemoattractant. Cells that reached the lower surface were stained with crystal violet. At least three independent experiments were performed with triplicates for each condition. Cells were counted in eight randomly selected microscopic fields. In the case of MDA-MB-231 cells, invasion was evaluated after 24 h.

### Migration assays

Migratory capacity of cells on ECM components fibronectin, laminin I, and type-I collagen was examined using the Radius™ 24-Well Cell Migration Assay kit (Cell Biolabs) following manufacturer's instructions. Briefly, 1,25 x 10^4^ cells were seeded per well and migration was monitored by time-lapse microscopy using a Zeiss Axio Observer Microscopy. Experiments were performed with triplicates and covered area was quantified at different times using Image J. Results were obtained after 24 h migration for MCF-7 cells and after 6 h for MDA-MB-231 cells.

### Mammospheres culture

4 x 10^4^ MCF-7 or 5 x 10^3^ MDA-MB-231 cells were plated in 6-well ultralow attachment plates (Costar) and grown in MammoCult Basal Medium (Stem Cell Research) supplemented with 10 % Mammocult Proliferation Supplement, 4 μg/mL heparine and 0.5 μg/mL hydrocortisone. After 7 days, mammospheres were collected and enzymatically dissociated as previously described [43]. Individual dissociated cells were cultured in 96-well ultralow attachment plates at a density of 20 cells/well. Mammospheres formation was monitored microscopically daily to ensure they derived from single cells and not from aggregates. Number of mammospheres was determined after 7 days culture.

### Animals and subcutaneous tumors

Four to five-week-old female athymic nude mice (*nu/nu* Swiss mice from Charles River Laboratories) were used for *in vivo* studies. To induce subcutaneous tumors, three groups of five mice were injected at one flank with 7 x 10^6^ cells of MCF-7-(fb), MCF-7-(ts) or MCF-7-(fb/ts) cells in 200 μL PBS. As a control, the opposite flank in each animal was injected with identical number of control MCF-7 cells transfected with an empty vector. Tumor growth was monitored weekly as previously described [29]. For estrogen supplementation, pellets of 1.5 mg 17-β-estradiol, 90-day release (Innovative Research of America), were implanted between the scapulae at the time of injection. Mice were housed under specific pathogen-free conditions and following the guidelines of the Committee on Animal Experimentation of the Universidad de Oviedo, Asturias, Spain.

### Human breast cancer tissue array

A breast cancer tissue array containing 16 tumors samples from different tumor stages (Acris Antibodies) was employed to evaluate *ADAMTS12* and *FBLN2* expression in human breast samples. Slides were processed for indirect peroxidase immunohistochemistry in the following way: sections were deparaffinized and rehydrated, and then rinsed in phosphate buffered saline (PBS) containing 1 % tween-20. For detection of fibulin-2 (H-250 antibody) and ADAMTS-12 (H-142 antibody), sections were heated in high pH Envision FLEX target retrieval solution at 80 °C for 20 min and then incubated for 20 min at room temperature in the same solution. Endogenous peroxidase activity (3 % H_2_O_2_) and non-specific binding (33 % fetal calf serum) were blocked and the sections were incubated overnight at 4 °C with primary antibodies described above using a 1:100 dilution for both antibodies. As secondary antibodies we used labelled polymer-HRP ready to use from DAKO. 3-3' diaminobenzidine was employed as a chromogen. Selected slides were counterstained with haematoxylin. The intensity of immunostaining was evaluated by two independent observers directly upon microscope and was scored as absent, slight, moderate and strong. Study methodologies were conformed to the standards set by the Declaration of Helsinki and were approved by the Ethics Committee of the Universidad de Oviedo-Principado de Asturias (Spain).

### Survival analysis

To assess the effect of ADAMTS-12 and fibulin-2 on breast cancer prognosis, survival probability was determined using the data available at www.kmplot.com [44]. 1592 patients were selected and we compared high (top 25 % of patients) and low (bottom 25 % of patients) expression of *ADAMTS12*, *FBLN2* and both genes simultaneously. Results were represented as Kaplan-Meier long-rank test survival plots.

### Statistical analysis

Statistical analysis were carried out using the GraphPad Prism 5.0 Software. Data were represented as means +/− S.E. The occurrence of significant differences was determined with the Student-Welch *t* test. *p* values under 0.05 were considered statistically significant (*p* < 0.05, *; *p* < 0.01, **; *p* < 0.005, ***).

## SUPPLEMENTARY FIGURES


















