# Supplementary material for: Interaction between the ADAMTS-12 metalloprotease and fibulin-2 induces tumor-suppressive effects in breast cancer cells
Source: Oncotarget. 2014 Jan 12;5(5):1253–64. doi: 10.18632/oncotarget.1690 (PMC4012729; doi:10.18632/oncotarget.1690)
Supplement: Supplementary file 1 [file oncotarget-05-1253-s001.docx]

**
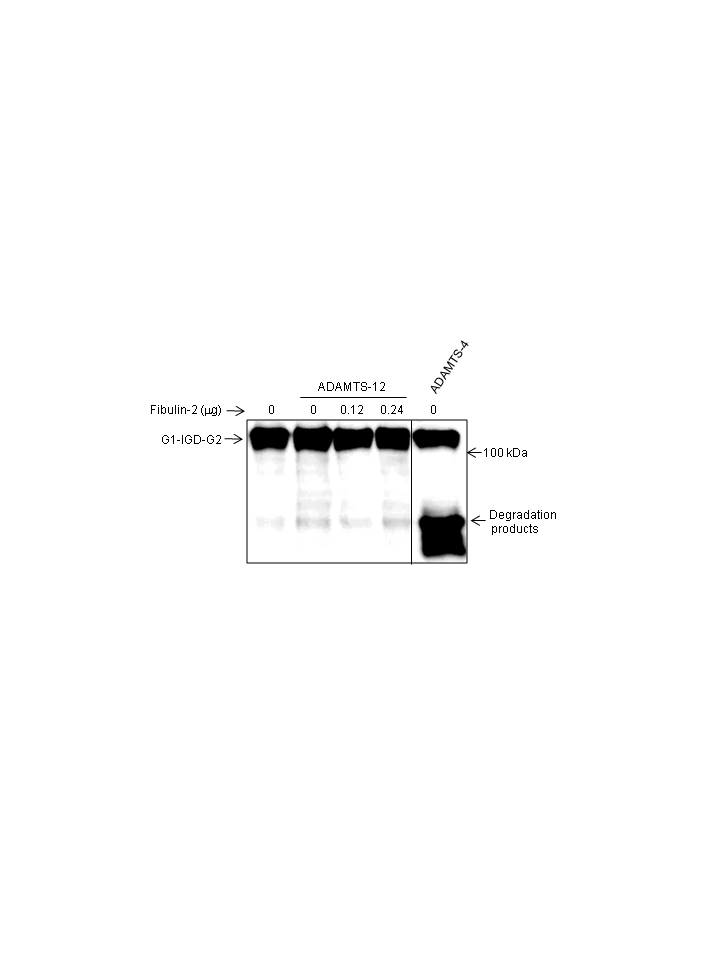
**

**Supplementary Figure S1**: **Fibulin-2 does not increased ADAMTS-12 enzymatic activity**. A peptide corresponding to the aggrecan IGD domain (G1-IGD-G2) was used as substrate. Two different amounts of fibulin-2, 0.12 and 0.24 μg, were employed. Degradation of same peptide by ADAMTS-4 was include as positive control. C, control sample lacking ADAMTS-12. Molecular weight marker is indicated on the right.

**Supplementary Figure S2**: **Relative expression levels of exogenous fibulin-2 and ADAMTS-12 proteins in extracts of transfected MCF-7 or MDA-MB231 cells**. Expression levels of fibulin-2, ADAMTS-12 or both proteins simultaneosuly in MCF-7 and MDA-MB-231 was independently quantified from western blots using the Image J software. Values for Fibulin-2 or ADAMTS-12 were normalized to actin levels within the same blot.

**
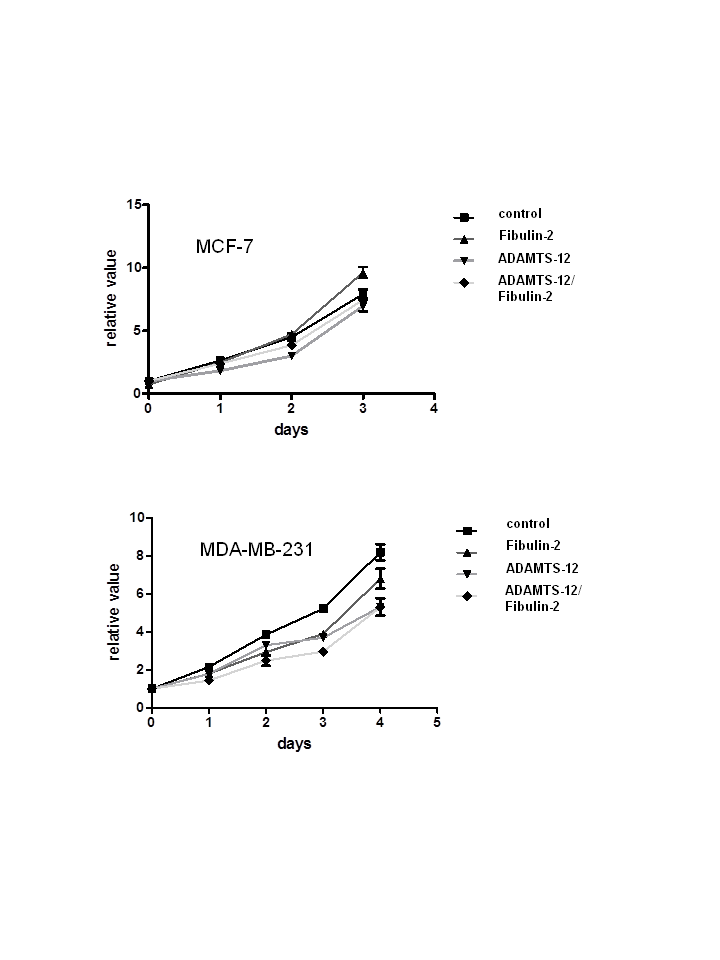
**

**Supplementary Figure S3: Cell proliferation of MCF-7 and MDA-MB-231 cells producing exogenous ADAMTS-12, fibulin-2 or both proteins simultaneously**. Cell proliferation rates were determined on three (MCF-7) or four (MDA-MB-231) consecutive days using an automated microtitre plate reader.

**
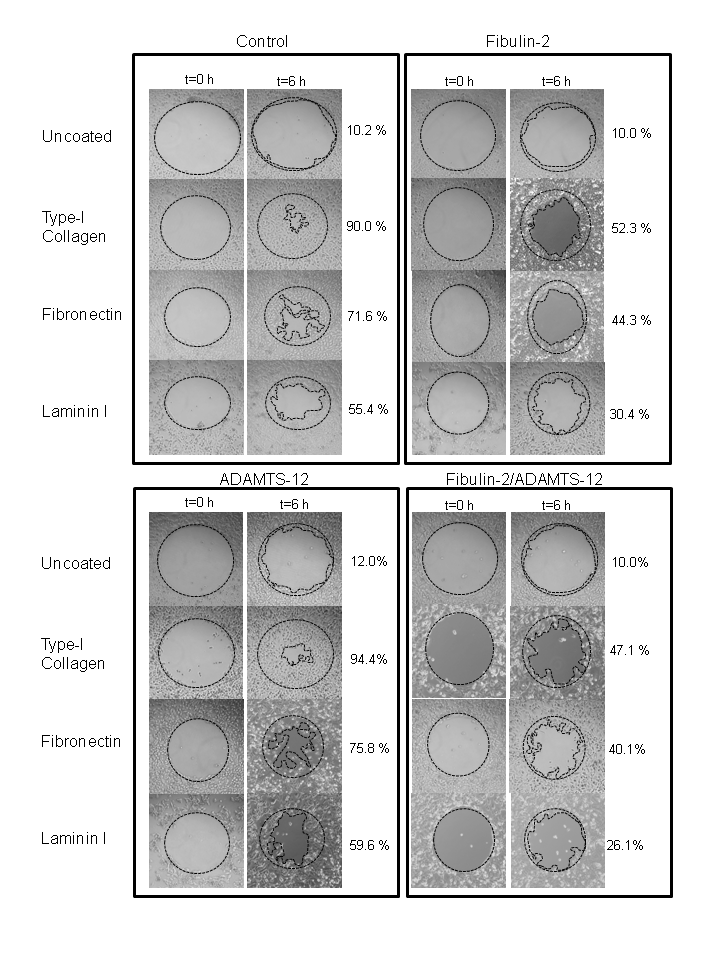
**

**Supplementary Figure S4: Interaction between fibulin-2 and ADAMTS-12 hinders migration of MDA-MB-231 cells on ECM components**. MDA-MB-231 cells producing fibulin-2, ADAMTS-12 or both proteins simultaneously were allowed to migrate in uncoated wells or wells coated with type-I collagen, fibronectin or laminin I. MDA-MB-231 cells transfected with an empty vector were used as control. Pictures of starting (t=0h) and final (t=6h) times are included. Starting point is indicated with a thin dotted line and final point with a thick dotted line. Percentage indicates the covered area after 6 h migration estimated from three different experiments.

**
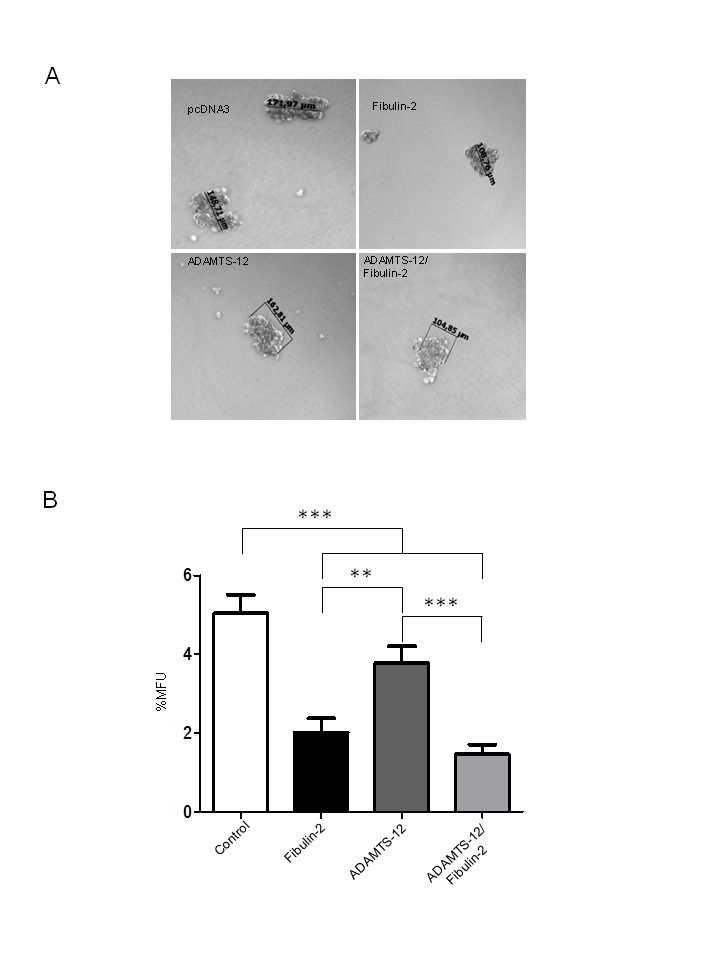
**

**Supplementary Figure S5: Reduction of self-renewal of mammosphere-forming units in MDA-MB-231 cells producing fibulin-2, ADAMTS-12 or both proteins**. (A) representative images of mammospheres derived from MDA-MB-231-(fn) (fibulin-2), MDA-MB-231-(ts) (ADAMTS-12), and MDA-MB-231-(fn/ts) (ADAMTS-12/fibulin-2). Control, MDA-MB-231cells transfected with an empty vector. Sizes of some mammospheres are indicated. (B) Mammospheres were dissociated and counted as indicated for MCF-7 cells.
